# Phase I trial of intravesical Suramin in recurrent superficial transitional cell bladder carcinoma

**DOI:** 10.1038/sj.bjc.6602650

**Published:** 2005-05-31

**Authors:** J J Ord, E Streeter, A Jones, K Le Monnier, D Cranston, J Crew, S P Joel, M A Rogers, R E Banks, I S D Roberts, A L Harris

**Affiliations:** 1 Department of Urology, Churchill Hospital, Oxford, UK; 2 Department of Medical Oncology, St Bartholomew's Hospital, London, UK; 3 Cancer Research UK Clinical Centre, St James's University Hospital, Leeds, UK; 4 Department of Cellular Pathology, John Radcliffe Hospital, Oxford, UK; 5 Growth Factor Group, Cancer Research UK, Weatherall Institute of Molecular Medicine, John Radcliffe Hospital, Headington, Oxford, UK

**Keywords:** bladder cancer, intravesical therapy

## Abstract

Suramin is an antitrypanosomal agent with antineoplastic activity, but with serious systemic side effects. We administered Suramin intravesically to determine a concentration with low toxicity but with evidence of a pharmacodynamic effect, to recommend a dose level for phase II trials. This was an open-labelled, nonrandomised dose-escalation phase I study. In all, 12 patients with a history of recurrent superficial bladder cancer were grouped into four dose levels (10–150 mg ml^−1^ in 60 ml saline). Six catheter instillations at weekly intervals were used. Cystoscopy and biopsy were performed before and 3 months after the start of treatment. Suramin was assayed using high-performance liquid chromatography, vascular endothelial growth factor (VEGF) using ELISA (enzyme-linked immunosorbent assay), and urinary protein profile using surface-enhanced laser desorption ionisation mass spectroscopy (SELDI). Minimal systemic absorption of Suramin was found at the highest dose of 150 mg ml^−1^. Urinary VEGF was affected by Suramin at doses above 50 mg ml^−1^, corresponding to the estimated threshold of saturation of Suramin binding to urine albumin. SELDI showed a specific disappearance of urinary protein peaks during treatment. Intravesical Suramin shows lack of toxicity and low systemic absorption. The results of this phase I trial support expanded clinical trials of efficacy at a dose of 100 mg ml^−1^ intravesically.

Bladder cancer has an incidence of 30/100 000 per year and three-quarters of patients present with superficial disease. In all, 30% of intermediate-risk superficial bladder tumours will recur within 2 years despite intravesical treatment with mitomycin C, and around 6% may progress to cause death within a median follow-up of 7 years (Tolley *et al*, 1996). The incidence of recurrence can be reduced with current intravesical regimens, with agents such as mitomycin C, epirubicin, or Bacillus Calmette Guerin (BCG). The drawback to current intravesical therapies is their toxicity and resistance to their effects.

Suramin is a polysulphonated naphthylurea and a potent antagonist of vascular endothelial growth factor (VEGF) (Bikfalvi *et al*, 1991). Suramin inhibits *in vitro* activity of several angiogenic factors produced by bladder cancer cell lines (Gansler *et al*, 1992; Walther *et al*, 1994) and cell proliferation. Suramin also inhibits basic fibroblast growth factor-induced angiogenesis *in vivo* in mice (Hosang, 1985; Coffey *et al*, 1987; Huang and Huang, 1988; Kopp and Pfeiffer, 1990; Fuller-Pace *et al*, 1991; Pesenti *et al*, 1992; Waltenberger *et al*, 1996), the activity of heparanase (Marchetti *et al*, 2003), and urokinase-type plasminogen activator (Marutsuka *et al*, 1995). We previously showed inhibition of the invasion of bladder cancer cell lines with Suramin in a bladder tissue explant model (Fujiyama *et al*, 2001).

VEGF is a rational target in superficial bladder cancer. We previously have shown that high VEGF levels in primary superficial tumours or in urine are related to early relapse and stage progression (O'Brien *et al*, 1995; Crew *et al*, 1996, 1997, 1999), and normal bladder, adjacent to the tumour, has VEGF levels much higher than nonmalignant controls. Since many recurrences are genetically identical to the primary, seeding is a possibility (Duggan *et al*, 2004) and prevention of vascularisation of each lesion may prevent recurrence and invasion.

When administered systemically, a wide range of serious side effects have been reported for Suramin including haematologic, ocular, metabolic (adrenal insufficiency; La Rocca *et al*, 1990), renal (acute failure; Figg *et al*, 1994) and neurological (Bowden *et al*, 1996). Suramin has a lengthy plasma half-life of approximately 40 days in man (Hawking, 1978), and renal clearance accounts for almost all the drug's elimination. There are a number of inherent advantages to the administration of Suramin intravesically. Owing to its relatively high molecular mass (1429 *vs* 334 for mitomycin C), systemic absorption should be very low. Additionally, in the plasma, 99.7% of Suramin is protein bound, which may interfere with its availability to bind growth factors. The much lower protein levels in urine could mean greater bioavailability. Graham reported excellent efficacy when given intravesically to rats in a chemically induced model of bladder cancer (Graham *et al*, 1995).

This study describes a phase I and pharmacological study of Suramin given intravesically to patients with bladder cancer. The primary objective of the study was to establish a recommended dose level of intravesical Suramin based on toxicity profile and pharmacodynamic end point of VEGF binding. The secondary objective was to investigate the effect of Suramin on bladder biopsies assessed by immunohistochemistry and to monitor global changes in urinary proteins using surface-enhanced laser desorption ionisation mass spectroscopy (SELDI).

## PATIENTS AND METHODS

### Eligibility

Patients for whom a rigid cystoscopy had been indicated following previous treatment for superficial bladder cancer or who had symptoms indicating a possible recurrence were included. Recurrences were detected using flexible cystoscopy. Entry criteria were that patients had to be 18 years or older, have a World Health Organization (WHO) performance status of 0–2, and have no toxic manifestations of previous treatments. Inclusion criteria were presence of Ta, T1, and Grade 1 or 2 multiple tumours (but not >7). Patients with single or >7 tumours, stage T2−4b, Grade 3 or carcinoma *in situ* (from four random bladder biopsies taken at pretreatment cystoscopy) were excluded. Concurrent treatment for other malignancies, prior radiotherapy, anticoagulant therapy, a history of adrenal insufficiency or steroid therapy were also exclusion criteria, as well as clinically significant hepatic or renal disease.

### Dose and dose escalation

Suramin was supplied by the NCI (USA), Division of Cancer Treatment and Diagnosis, in lyophilised form as a powder stored at room temperature. Suramin is freely soluble in water. The dose was diluted in 60 ml normal saline for each intravesical instillation. Patients were grouped into four treatment levels, with intergroup dose escalation as the trial progressed. Significant, that is, Grade 3 (NCI Common Toxicity Criteria), toxicity would prevent progression to the next treatment level and expand recruitment to a maximum of six patients at that level. Dose levels are described in Table 1.

### Study design

The protocol was approved by the CRC (Cancer Research Campaign) Protocol Review Committee, the CIRB (Central Institutional Review Board), and the Local Research Ethics Committee (LREC). The study was carried out in accordance with the Declaration of Helsinki. Written informed consent was obtained. This was an open-labelled, nonrandomised phase I study with an intended recruitment of 12 patients. Pretreatment evaluation included full history, physical examination, and assessment of WHO performance status. In addition, a complete blood count, clotting screen, renal, and liver biochemical profiles and urine analysis were performed. These were repeated weekly and 3 weeks after the end of treatment. At the pretreatment transurethral resection of bladder tumour (TURBT), random biopsies of normal bladder and samples of the tumour were taken, and both were fixed in formalin and paraffin embedded.

Patients received their first instillation of Suramin at least 14 days postcystoscopy and TURBT. This was to allow the bladder to heal and to reduce systemic absorption. The drug was then given once a week for 6 weeks. The bladder was drained prior to and postinstillation. Treatments were left *in situ* for 2 h, during which time the patient remained horizontal, and was asked to turn through 90° every 15 min.

At 3 weeks after the end of the treatment, a further cystoscopy was performed under general anaesthetic and further random biopsies taken with tumour resection if necessary.

### Sample handling

Blood samples were collected in EDTA tubes and centrifuged at 2000 **g** for 10 min within 30 min of sampling. The plasma was stored in aliquots at −70°C. The volume of urine drained from the bladder at the end of 2 h was measured. An aliquot of 10 ml of urine was collected, 1 ml of protease inhibitor was added (Sigma P2714, Protease Inhibitor Cocktail containing AEBSF, bestatin, EDTA, E-64, leupeptin, and aprotinin), and the urine was centrifuged at 2000 **g** for 10 min within 30 min of sampling. Aliquots were stored at −70°C. It has previously been shown that protease inhibitor does not affect VEGF results (Crew *et al*, 1999).

### Suramin pharmacokinetics

Suramin concentration in plasma and urine was determined using a high-performance liquid chromatography (HPLC) assay following protein precipitation. Briefly, 200 *μ*l of 1 M tetrabutyl ammonium bromide (ion-pairing agent) and 200 *μ*l 10 *μ*g ml^−1^ methylphenyl-phenyl-hydantoin (internal standard) were added to a 250 *μ*l sample (plasma or urine). Proteins were then precipitated with 500 *μ*l methanol by incubation at 4°C for 30 min, after which the supernatant was injected into the HPLC system. Standard curves covered the range of 0–30 *μ*g ml^−1^ plasma and 0–200 *μ*g ml^−1^ urine. Urine samples were diluted by up to 900-fold (depending on the dose level) with water to ensure the concentration was within the standard range. Separation of peaks of interest was achieved using a 5 *μ*M Hypersil C_18_ column (Jones Chromatography, Hengoed, Wales) and a mobile phase comprising 10 mM phosphate buffer (10 mM disodium hydrogen phosphate and 10 mM sodium dihydrogen phosphate, pH 7.5), with 4 mM tetrabutylammonium bromide and 52.5% methanol. Eluting peaks were monitored at 230 nM by UV detection. The limit of quantitation was 0.5 *μ*g ml^−1^ plasma and 5 *μ*g ml^−1^ urine. Reproducibility (covariance) of quality control samples at 0.5, 4, and 20 *μ*g ml^−1^ plasma and 5 and 50 *μ*g ml^−1^ urine was <12%. This protocol released Suramin from protein and measured total Suramin.

### Pharmacodynamics

VEGF was measured in the urine and plasma using a standard enzyme-linked immunosorbent assay (ELISA) (R&D Systems) before each Suramin dose and was compared with that detectable after each Suramin dose. This ELISA has previously been tested for use with urinary samples for recovery of VEGF from urine (Crew *et al*, 1999). Minimum detectable dose of VEGF by this ELISA is typically less than 5 pg ml^−1^.

To assess the general effect of Suramin instillation on global urine protein profiles, urine samples were also analysed using SELDI (Ciphergen, USA) with the SAX2 and H4 chip surfaces. The instrument was calibrated at the start and end of the run using bovine ubiquitin (MW 8564.8), bovine superoxide dismutase (MW 15591.4), and bovine *β*-lactaglobulin A (MW 18 363.34). Samples were loaded using a bioprocessor as described previously (Rogers *et al*, 2003), but for SAX chips, the buffer used for binding, dilution, and washing was 20 mM sodium phosphate, pH 8.0/0.1% (v v^−1^) NP40 and for the H4 chips it was prewet with 50% acetonitrile with 10% acetonitrile/0.1% TFA being used for wash steps. Additionally, in each case, sample loading was standardised on the basis of creatinine with a diluted volume of urine equivalent to a final creatinine concentration of 2.5 mM being loaded in a 50 ml volume. Samples were analysed using parameters of a high mass of 100 kDa, optimum mass range 3–20 kDa, laser intensity of 210, sensitivity of 10 and collecting 50 transients across the spot surface. This was carried out on samples pre- and post-Suramin dose 1, pre- and postdose 5 or 6, and on the 4 weeks post-treatment samples for each of three patients (patient 7 at dose level 2 and two patients 9 and 12 at dose level 3). Samples were all analysed on the same batch of chips on a single day to minimise variability.

### Immunohistochemistry

Paraffin sections of paired normal bladder available from 11 patients were heated to 60°C for 15 min, dewaxed in citrate, and rehydrated by passage through graded alcohols. Sections of normal bladder were stained for VEGF bound to KDR (VEGFR2) receptor using 11B5 monoclonal antibody (East Coast Biologics Inc., North Berwick, ME, USA). (Brekken *et al*, 1998). Antigen retrieval was carried out by pressure-cooking for 3 min in 0.01 M citrate, pH 6.0. Primary antibody was applied (1 : 4 in 1 × TBS) for 1 h. Control slide used was normal human tonsil where staining of vessels, stromal cells and luminal surface was seen. Labelling was detected using anti-mouse system (Dako) as per the manufacturer's instructions. Sections were then counterstained with haematoxylin and mounted. Staining of blood vessels was performed with anti-CD34 (QBEnd 10 antibody) at a final concentration of 1 : 100 using a similar protocol. The number of CD34-stained microvessels was counted in the lamina propria immediately adjacent to the urothelium on a high-power (× 40 objective) lens and the proportional vascular space volume determined using a Chalkley point graticule (Chalkley, 1943). Between 5 and 10 adjacent high-power fields were counted for each specimen. The highest three values were taken as hotspots and the mean was taken.

## RESULTS

In all, 12 patients were entered into the study, all of whom were evaluable for toxicity. Their details are shown in Table 2.

### Toxicity

Three patients experienced minor rises in fasting blood glucose (insufficient for a diagnosis of diabetes mellitus) and one patient developed minor lymphopenia at the lowest dose level, of unproven significance. No serious adverse events were noted and no patients complained of urinary symptoms related to treatment. In fact, two patients previously with nocturnal frequency of micturition noted a marked improvement in this symptom while receiving treatment. There were no drug-related adverse events above Grade 1.

### Clinical outcome

Three patients had recurrent tumours and two had dysplasia at the final cystoscopy. Four of these were in the lower two dose groups (4/6 *vs* 1/6 Fisher's exact test not significant *P*=0.2).

### Urine pharmacokinetics

**Figure 1:**
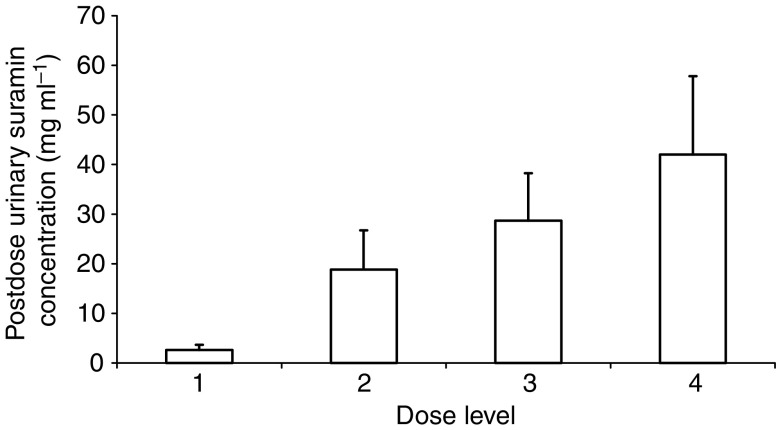
Mean postdose urinary Suramin concentrations by dose level (see Table 1). This represents the concentration of urine drained from the bladder at the end of the 2-h instillation. Error bar equals 1 s.d. Differences between groups are highly significant (two-tailed *t*-test, variances not assumed to be equal *P*<0.01).

All urine samples immediately before preinstillation had undetectable levels of Suramin. Urine drained at the termination of each instillation contained levels as shown in Figure 1. There was a clear relationship between urinary Suramin concentration and dose with mean levels of 28.7 and 42.0 mg ml^−1^ at the two highest levels. Repeat analysis of urine samples from four patients for internal consistency showed that in all cases the final results were within 5% of one another. From this evidence, dilutional or assay error accounts for <5% deviation between results. The calculated total amount of Suramin recovered at the end of the instillation for four complete treatment courses was 87% (s.d. 28%).

### Plasma pharmacokinetics

In all, 12 patients were evaluable for pharmacokinetics. All plasma samples for patients up to and including level 3 had no detectable Suramin, that is, less than 0.5–6 *μ*g ml^−1^. Two patients at dose level 4 had detectable Suramin levels above 6 *μ*g ml^−1^. These were in patient 11 after instillations 5 and 6 with levels of 6.3 *μ*g ml^−1^, 1163 *μ*g ml^−1^, respectively, and in patient 12 after instillation 5 with a level of 333 *μ*g ml^−1^. High levels were diluted and rerun, with the repeat confirming the value in both cases. No clinically traumatic catheterisation occurred during the study. These patients were not receiving any different drugs on these days compared to other days.

### Pharmacodynamics: plasma and urine VEGF

**Figure 2:**
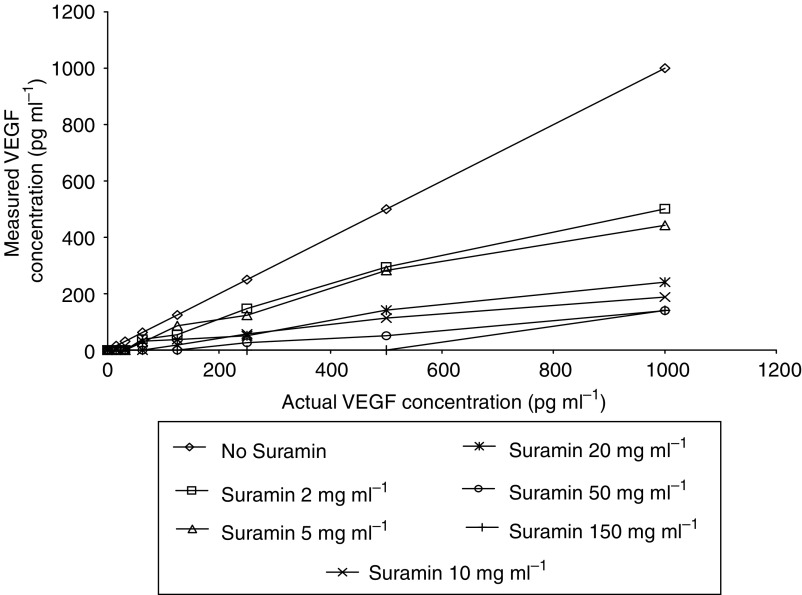
Suramin interference with VEGF ELISA. Standard concentrations of VEGF were spiked with different levels of Suramin, and then the VEGF was measured.

Suramin shows high affinity for the heparin-binding growth factor VEGF. Standard samples of VEGF were spiked with increasing concentrations of Suramin, with resulting interference of the VEGF ELISA as shown by the poor recoveries in Figure 2. The ELISA is inhibited by only small concentrations of Suramin, for example, 2 mg ml^−1^ causing an approximate 40% fall in the measured level of VEGF at all concentrations, while 150 mg ml^−1^ caused almost a 100% fall at all concentrations compared with control. A number of means were tried to disrupt the interaction between Suramin and VEGF. These included dialysis through a semipermeable membrane, passage through an ion-exchange column (Suramin having a negative charge), dissolution in 4 M NaCl, and the addition of 10% SDS followed by boiling. None of these methods had any effect on the VEGF–Suramin binding as measured by ELISA (data not shown). A similar effect was demonstrated using spiked human urine samples; thus, in the data that follows, VEGF levels must be interpreted in the light of measured Suramin concentrations and reflect free VEGF concentrations.

In all, 12 patients were evaluable for pharmacodynamics. Plasma VEGF results did not change significantly over the course of treatment at any dose level. Plasma levels of VEGF were in the normal range for all patients, except patient number seven, who for unknown reasons had persistently raised levels.

**Figure 3:**
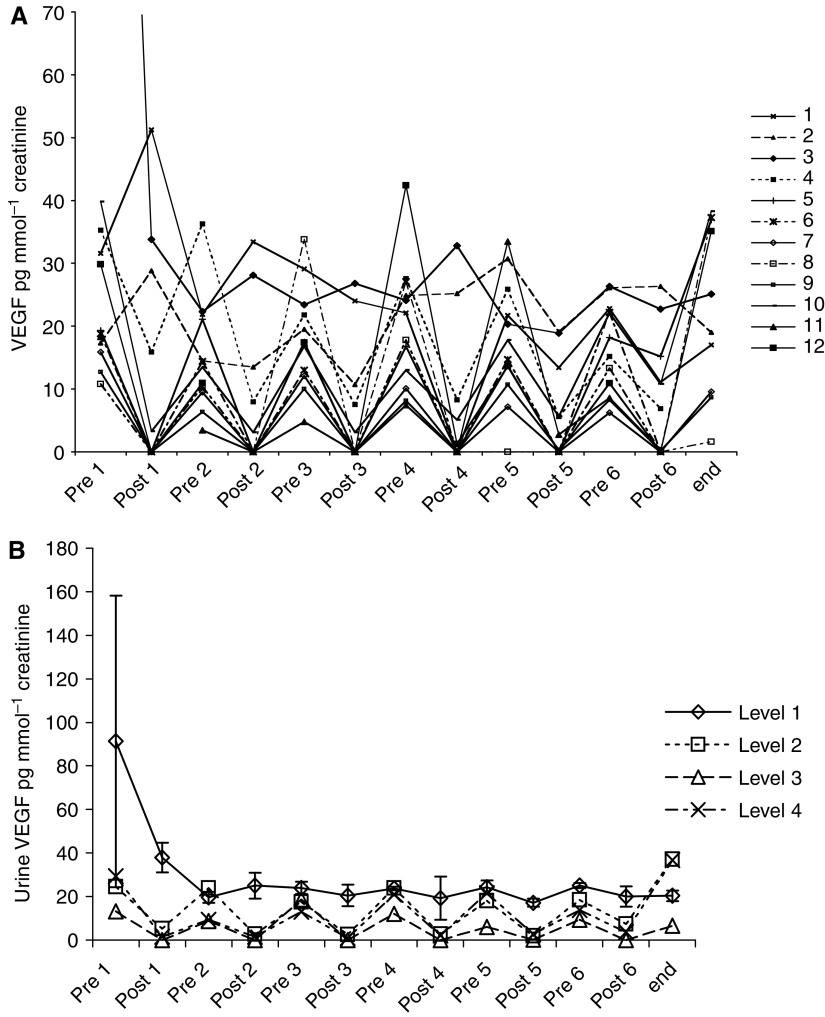
(**A**) Urine VEGF pre- and postdose by individual patient. Individual patients are indicated by a number from 1 to12. (**B**) Urine VEGF pre- and postdose grouped by dose level of Suramin. Three patients per level; difference between level 1 and other levels *P*⩽0.05 is seen at postdoses 1, 2, 3 and 5. Error bars equal s.e.m.

Urinary VEGF was quantified by ELISA with values normalised to urinary creatinine, to adjust for variation in urine concentration (Figure 3A). Mean VEGF levels immediately before each treatment and at the end of the treatment course are shown by dose cohort (Figure 3B). The measurement of urinary VEGF levels was profoundly affected by the presence of Suramin, especially at doses above level one. This corresponds to the previously demonstrated inhibition of the VEGF ELISA by concentrations of Suramin as low as 2 mg ml^−1^. Suramin did not alter urinary VEGF over the course of treatment on average, as assessed by the baseline value before each instillation.

### Surface-enhanced laser desorption/ionisation

**Figure 4:**
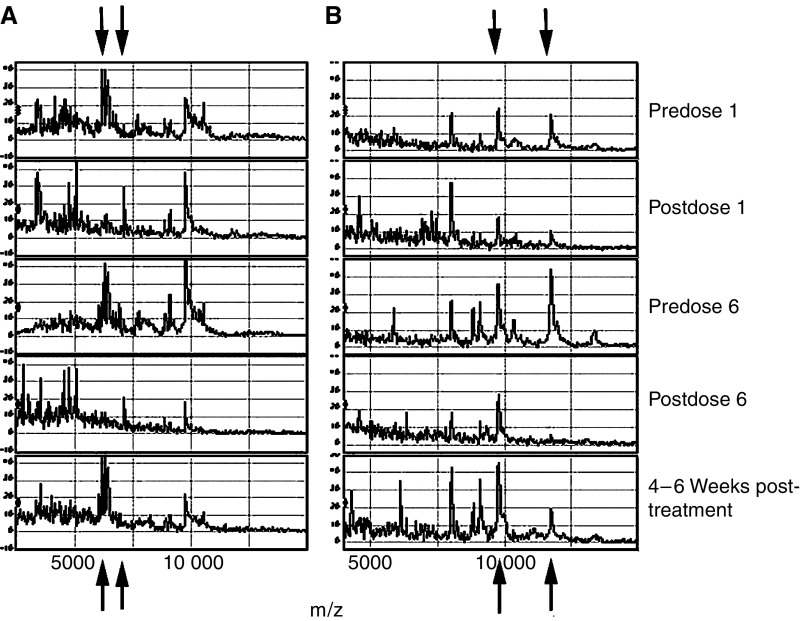
SELDI profiles of urine samples from patient 9 on a SAX2 chip (**A**) and patient 12 on an H4 chip (**B**). Pre- and postdose 1 and 6, and 4 weeks postcessation of Suramin treatment. Arrows identify peaks that are either lost or gained.

Although the convention with urine samples is to normalise results for urine creatinine to correct for hydration status, differences in protein loading on SELDI chips can influence the profile (Rogers *et al*, 2003). For comparison of profiles, samples ideally should be loaded normalised for protein, therefore (Rogers *et al*, 2003). However, in this study, it proved impossible to assay protein concentration due to the interference of Suramin. Therefore, loading was normalised by creatinine, which should allow intrapatient comparison. Generally, the profiles on both the CM10 and H4 chips post-Suramin treatment showed a marked loss or reduction of peak number (Figure 4). However, it was noticeable that not all peaks were affected to the same extent with the peak at 9732.7–9741.2 with both the SAX2 and H4 chips being little affected. Conversely, in patients 9 and 12, the appearance of peaks at 4746.0–4749.2 and 5063.8–5069.2 on the SAX2 chips was noted with Suramin treatment. Although not examined under optimal higher laser intensities, similar loss of peaks was also seen in the higher mass region, although far fewer peaks are seen there. The profiles generated 4 weeks postcessation of treatment resembled the pretrial profiles.

### Immunohistochemistry

In all, 22 normal bladder biopsies from 11 patients pre- and post-treatment were available for staining. Mean microvessel count in pretreatment samples was 141 mm^−3^ (s.d. 45) and in post-treatment 140 mm^−3^ (s.d. 56), and mean proportional vascular volume estimated using the Chalkley random point graticule was 17% (s.d. 5.2) and 18% (s.d. 3.0), respectively (NS paired *t*-test).

**Figure 5:**
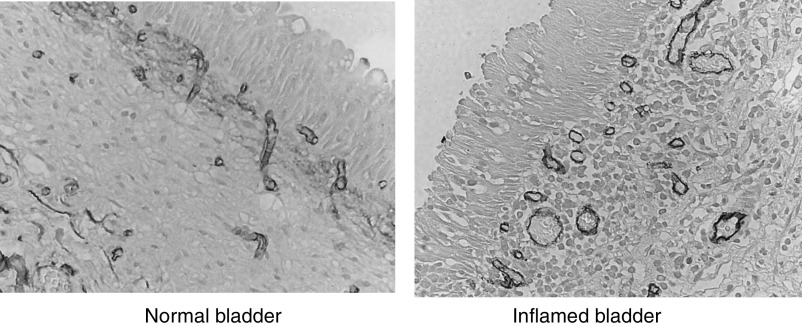
Staining for blood vessels in inflamed and noninflamed bladder with antibodies to CD31.

Mean microvessel count in severely inflamed samples (*n*=4, mean 186 mm^−3^, s.d. 66) was significantly higher (*P*=0.04) than in mildly or noninflamed samples (*n*=18, mean 131 mm^−3^, s.d. 42) and there was a trend to higher proportional vascular volume in severely inflamed samples (mean 23%, s.d. 5.2% *vs* mean 16%, s.d. 2.9%, *P*=0.07). The number of inflamed samples in pre- and post-treatment groups was not significantly different: two severely inflamed samples in each group, overall seven biopsies reported as any degree of inflammation in pretreatment group, and nine inflamed in post-treatment. Figure 5 illustrates typical staining patterns seen in the inflamed and the noninflamed bladder.

VEGF staining was scored on the basis of intensity and breadth of staining of the urothelium. Positive was defined as a biopsy showing full thickness epithelial staining either in patches or along the entire epithelial border. All biopsies showed staining of the umbrella layer of the urothelium, but there was no significant difference between pre- and postbiopsy samples in full thickness staining: four of 11 pretreatment biopsies stained positive and two of 11 post-treatment stained positive. Three of nine noninflamed and three of 13 inflamed were positive. Neither difference was significant (*χ*^2^ test).

## DISCUSSION

This trial has shown low toxicity, and the low systemic absorption of Suramin occurred only at the highest dose. Dose determination principally rests on the interpretation of pharmacodynamic data concerning the efficacy of Suramin in inhibiting the action of heparin-binding growth factors as surrogate markers of activity.

Suramin greatly interfered with the ELISA quantification of VEGF in urine. This inhibition of protein–protein interaction on which the ELISA depends is likely to be due to free Suramin binding VEGF and preventing antibody attachment to its antigen. Binding of Suramin to albumin, the main protein in the urine, will be dependent on their relative concentration ratio, as determined previously (Roboz *et al*, 1998). When Suramin is in excess of albumin, as in the vast majority of cases here, the number of Suramin molecules bound to each albumin rises rapidly, up to a maximum of 20 Suramin per albumin. Assuming albumin concentrations in urine up to a normal range of 20 mg l^−1^, up to 10 *μ*g ml^−1^ Suramin may be bound. Once this threshold is exceeded, inhibition of urinary VEGF quantification by ELISA occurs. This end point was considered a desirable biological effect to assess the ability of the free Suramin to inhibit a high-affinity specific interaction of VEGF with a binding site.

This was found in all cases at dose level 2 (50 mg ml^−1^ Suramin instilled), Figure 3B. The mean postdose urinary Suramin concentration measured at this level was 18 mg ml^−1^ (Figure 1). The one patient in whom VEGF quantification was not completely inhibited at this level was the one who produced the most urine during the 2-h instillation period; mean Suramin concentration was 11 mg ml^−1^. Full inhibition of the VEGF ELISA was only attained in patients treated with level 3 dosing (100 mg ml^−1^ Suramin instilled) in whom mean postdose urinary Suramin concentration was 29 mg ml^−1^. Of particular note, this is 300 times higher than the concentration required to inhibit transitional cell carcinoma cell growth in culture (Gansler *et al*, 1992).

One other phase I trial of intravesical Suramin has been published (without pharmacodynamic data) with the majority of the nine patients receiving between 0.3 and 154 mg ml^−1^ dose in 60 ml of normal saline over a 6-week period (Uchio *et al*, 2003). Bladder spasms associated with treatment occurred only in two patients with concentrations over 300 mg ml^−1^. Systemic absorption was <40 *μ*g ml^−1^ in all patients.

In our study, two treatments at the highest dose level of 150 mg ml^−1^ demonstrated high systemic absorption (334 and 1163 *μ*g ml^−1^ plasma level postdose). There was no associated clinical adverse effect. Plasma levels above 275 *μ*g ml^−1^ when maintained for over 4 weeks by intravenous infusion cause clinically significant neurological adverse effects such as paresis (Bowden *et al*, 1996). With a long half-life of 40 days, toxicity from a one-off level of 334 *μ*g ml^−1^ could potentially be significant. Plasma Suramin returned to <1 *μ*g ml^−1^ 1 and 3 weeks (respectively) after these high levels were detected, suggesting that these represent transient peaks and do not produce a reservoir.

SELDI provides a semiquantitative means of profiling, although many factors may affect the results including ion suppression. The general trend was of less protein peaks in the urine samples post-Suramin, although not all proteins were affected to the same extent. This was also seen if Suramin was spiked into urines *in vitro* and therefore is unlikely to reflect effects of Suramin on release of protein by bladder cells. Suramin, which is negatively charged, may compete with proteins binding to the chip surface with selective effects due to relative binding affinities. Alternatively, binding of Suramin to specific proteins may either alter their binding properties or cause their aggregation.

Three patients had recurrent tumours and two had dysplasia at the final cystoscopy. Four of these five recurrences were in the lower two dose groups. These numbers are insufficient to reach statistical significance, but the trend to fewer recurrences in the higher dose groups supports the laboratory end point determination.

In conclusion, intravesical Suramin is well tolerated in the treatment of patients with recurrent superficial bladder cancer. Levels are undetectable in the blood until the intravesical dose reaches 150 mg ml^−1^. Inhibition of the VEGF ELISA occurred reliably in patients at the 100 mg ml^−1^ level but not at lower levels. These results support the progression to expanded clinical trials of efficacy using a dose of 100 mg ml^−1^ intravesically.

Our *in vitro* bladder explant model (Fujiyama *et al*, 2001) suggested that Suramin very effectively inhibits implantation; therefore, the most effective time for Suramin administration may be at the time of resection as demonstrated with mitomycin C (Tolley *et al*, 1996). In our study, the first instillation was 14–18 days postresection to allow the bladder to heal and reduce systemic absorption. However, the low toxicity demonstrated and the recovery of urine VEGF levels after 1 week suggest that one or more doses of Suramin could be given at the time of resection and within the first 2 weeks. Theoretically, the healing bladder wound might secrete high levels of VEGF or heparin-binding growth factors encouraging tumour cell survival. Suramin's effect on superficial tumour itself was not assessed in this study; a study utilising a marker lesion would be better placed to do that.

## Figures and Tables

**Table 1:** Suramin dose levels *vs* treatment group

**Dose level**	**Concentration of Suramin (mg ml^−1^)**	**Total dose (mg)**
1	10	600
2	50	3000
3	100	6000
4	150	9000

**Table 2:** Details of the patients entered into the Suramin trial, also showing histology of recurrences

**Patient number**	**Dose level**	**Dose level (mg ml^−1^)**	**Number of instillations**	**Age (years)**	**Gender**	**Histology**	**Recurrence post-treatment**
1	1	10	6	71	Female	G2/T1	G2/T1
2	1	10	6	61	Male	G2/Ta	
3	1	10	6	61	Male	G2/T1	Dysplasia
4	2	50	6	65	Male	G1/Ta	
5	2	50	6	57	Male	G2/Ta	G2/Ta
6	2	50	6	81	Male	G2/Ta	Dysplasia
7	3	100	6	64	Male	G2/Ta	
8	3	100	6	52	Female	G1/Ta	
9	3	100	6	59	Male	G1/Ta	
10	4	150	6	69	Male	G2/Ta	
11	4	150	6	63	Female	G1/Ta	G1/Ta
12	4	150	6	62	Male	G1/Ta	
